# Residential elevation and its effects on hypertension incidence among older adults living at low altitudes: a prospective cohort study

**DOI:** 10.1265/ehpm.22-00001

**Published:** 2022-05-03

**Authors:** Wensu Zhou, Wenjuan Wang, Chaonan Fan, Fenfen Zhou, Li Ling

**Affiliations:** Department of Medical Statistics, School of Public Health, Sun Yat-sen University, Guangzhou, China

**Keywords:** Older adults, Altitude, China, Hypertension, Prevention

## Abstract

**Background:**

Research on the relationship between residential altitude and hypertension incidence has been inconclusive. Evidence at low altitudes (i.e., <1,500 m) is scarce, let alone in older adults, a population segment with the highest hypertension prevalence. Thus, the objective of this study is to determine whether hypertension risk may be affected by altitude in older adults living at low altitudes.

**Methods:**

This prospective cohort study collected data from the Chinese Longitudinal Healthy Longevity Survey (CLHLS). We selected 6,548 older adults (≥65 years) without hypertension at baseline (2008) and assessed events by the follow-up surveys done in 2011, 2014, and 2018 waves. The mean altitude of 613 residential units (county or district) in which the participants resided was extracted from the Digital Elevation Model (DEM) of the National Aeronautics and Space Administration (NASA) and was accurate to within 30 m. The Cox regression model with penalized splines examined the linear or nonlinear link between altitude and hypertension. A random-effects Cox regression model was used to explore the linear association between altitude and hypertension.

**Results:**

The overall rate of incident hypertension was 8.6 per 100-person years. The median altitude was 130.0 m (interquartile range [IQR] = 315.5 m). We observed that the exposure–response association between altitude and hypertension incidence was not linear. The shape of the exposure–response curve showed that three change points existed. Hypertension risk increased from the lowest to the first change point (247.1 m) and slightly fluctuated until the last change point (633.9 m). The risk decreased above the last change point. According to the categories stratified by the change points, altitude was only significantly associated with hypertension risk (hazard ratio [HR] = 1.003; 95% confidence interval [CI] = 1.002–1.005) under the first change point (247.1 m) after adjusting for related covariates.

**Conclusion:**

Our study found that the association between altitude and hypertension risk might not be linear. We hope the further study can be conducted to confirm the generality of our findings.

**Supplementary information:**

The online version contains supplementary material available at https://doi.org/10.1265/ehpm.22-00001.

## Background

Hypertension is one of the most common chronic disorders in older adults, and its prevalence increases with age. It is a leading risk factor for cardiovascular diseases (CVDs) and premature death worldwide [1]. Alongside the dramatic rise in aging populations and an increasing global disease burden of CVDs [2, 3], hypertension has become a serious public health problem in almost all countries.

Hypertension is a complex clinical syndrome that is associated with lifestyle, genetics, and mental stress. With advancing understanding of the risk factors for hypertension, environmental factors have attracted increasing attention in recent research [3], one of them being residential altitude. On the one hand, experts found that the prevalence of hypertension was higher in populations that resided in high altitudes than in those residing in low and moderate altitudes, where high altitudes were defined as elevations as low as 1,500 m to those as high as 8,800 m [4–7]. On the other hand, several observational and clinical studies have reported a significant correlation between hypertension risk and residential altitude [8–10]. For instance, one meta-analysis summarized studies conducted in Tibet (3,000–4,000 m) and reported that each 100-m increment in altitude was associated with a pooled 2% increase in hypertension risk [11]. A study conducted in Argentina indicated that each unit increment in altitude had induced a 10.55 and 6.27 mm Hg rise in systolic blood pressure (SBP) and diastolic blood pressure (DBP), respectively, among 185 children aged 5–14 years [12]. Another study conducted among 152 children also showed that a high-altitude location was associated with a 17.44 mm Hg increase in blood pressure (BP) [13]. However, existing epidemiological surveys have also demonstrated contradicting conclusions [14, 15]. According to a study conducted by Handler et al., low altitude (i.e., ≤1,500 m) was also positively associated with elevated BP [10]. A survey conducted in Peru showed that residing in high-altitude locations would reduce the risk of hypertension by approximately 26% compared with residing in low- and moderate-altitude locations [9]. Several studies have provided possible explanations for these inconsistencies. Biologically, hypoxia leads to deficiencies in the blood supply in the body, raising the risk of hypertension [10]. The effects of altitude on hypertension may in turn be offset by people’s adaptive capacities [16]. Thus, the existence of an altitude threshold over which hypertension risk increases and under which the risk decreases is plausible. However, the existence of altitude thresholds remains unknown.

Furthermore, previous studies have suggested several biases. First, most of these studies have been conducted in high-altitude areas [13], leading to a lack of data from low altitude locations. However, low altitudes are more likely to have higher population densities, which should warrant more attention. Let alone, Pei et al. also showed that every 200-m rise in altitude above sea level had an underlying effect on the hypertension incidence [8]. Second, surveys using large-scale (i.e., national-level) altitude estimations are still limited [7]. In addition, few prospective cohort studies and long-term evaluations of the effects of altitude on hypertension have also limited the ability to verify and generalize related conclusions [7, 17]. Finally, most previous studies have been conducted among the following populations: children, pregnant women, and young or middle-aged participants [13, 17, 18]. While the physiological characteristics of older individuals were indicated in these previous studies, that is, they were more severely impacted by environmental factors due to age-related organ function decline [19]. Therefore, given the significant and complex correlation of altitude as an environmental factor with hypertension incidence and the relatively high hypertension prevalence in older adults, more studies to evaluate the association between low altitude and hypertension risk are warranted. In particular, further understanding of this link in older individuals may help in designing and implementing more effective prevention strategies in the future.

The main objectives of this study were as follows: (i) to explore the linear or nonlinear association between altitude and hypertension incidence in adults aged ≥65 years based on a prospective cohort study of older individuals living at low altitudes, and (ii) to identify the impact of altitude exposure on hypertension risk. We expect that our findings may help healthcare providers accurately identify the risk of hypertension.

## Methods

### Study population

This was a prospective, cohort study that explored the effect of altitude on hypertension based on an open public database, the Chinese Longitudinal Healthy Longevity Survey (CLHLS). This was a national-level investigation that recruited participants aged ≥65 years from 23 of the 31 provinces/autonomous regions/municipalities in China. A total of 85% of the population in mainland China resides in these provinces, autonomous regions, and municipalities. The project was established in 1998, and follow-up investigations were conducted in 2000, 2002, 2005, 2008, 2011, 2014, and 2018. The introduction of the CLHLS has been previously described in detail [20].

**Fig. 1:**
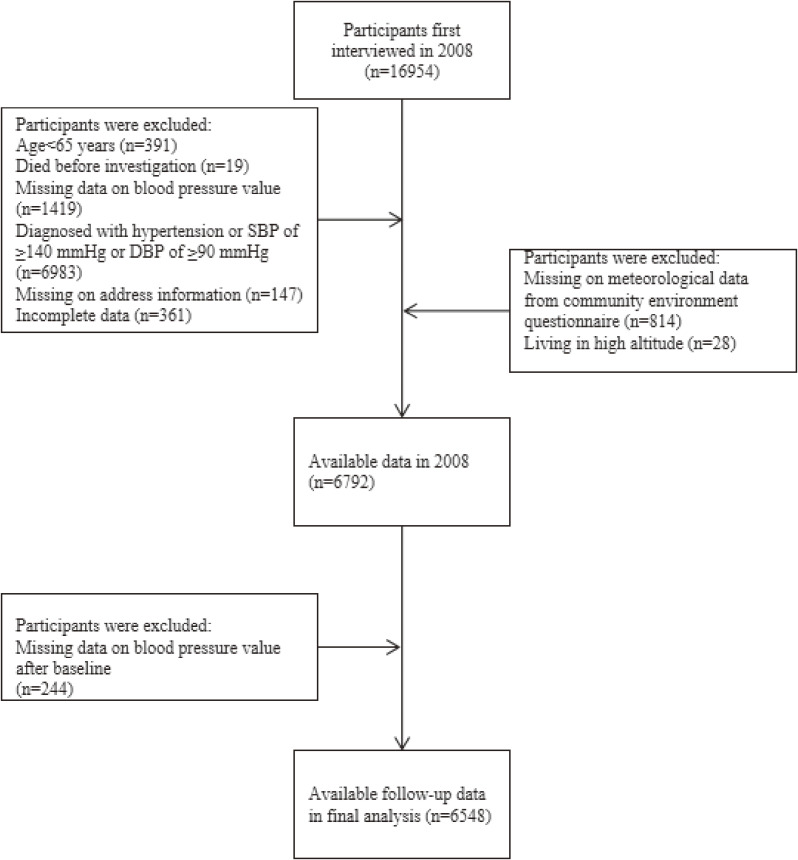
Selection strategy of the study participants. The flowchart depicts how participants from the Chinese Longitudinal Healthy Longevity Survey (2008–2018) were selected for this study. Abbreviations: SBP, systolic blood pressure; DBP, diastolic blood pressure.

The current study consisted of participants who were first recruited in 2008 and assessed events by the follow-up surveys done in 2011, 2014, and 2018 waves. Due to the privacy protection provided by the CLHLS, the accurate home addresses of the participants were not available; we could only acquire the county (district) name of residence from the community environment questionnaire in 2008, 2011, and 2014 waves [21]. Participants who were diagnosed with hypertension (i.e., SBP of ≥140 mm Hg, DBP of ≥90 mm Hg, or a normal BP but diagnosed with hypertension by a public hospital) and had missing demographic information, BP measurements, geographical information, and other relevant variables were excluded in the study. Finally, the study included 6,548 participants from 613 residential units (county or district), with complete baseline and follow-up interview information. Figure 1 shows the selection strategy of the study participants.

We confirmed that the residential units of participants in this cohort were relatively fixed because older adults were less likely to move to another address, as well as the influence of the strict Hukou registration management system on migration in China. In our study, we found that 156 (2.4%) older adults changed their residences (county/district/rural/urban level) during follow-up. In line with a previous study [22], we excluded these participants in the sensitivity analysis. In this study, the detailed date of hypertension incidence was not clear because the follow-up examinations occurred every 2–3 years for each cohort, we could only determine the results of each follow-up. Thus, the endpoint event was incident hypertension, and censoring included death, loss to follow-up, and end of follow-up. The duration of follow-up was calculated from the interview date of the baseline survey to that of the last interview or administrative censoring date (i.e., the middle date between the last survey when the participant was interviewed and the subsequent survey, for participants who were lost to follow-up) and was presented as person-years (days/365).

Informed consent was obtained from each participant before the investigation. The research ethics committees of Peking University and Duke University granted approval for the Protection of Human Subjects for the study, including for data collection.

### Hypertension assessment

BP measurement was a part of the individual questionnaire in each interview. Each participant underwent two BP measurements after at least five minutes of rest. A mercurial sphygmomanometer (upper arm type; Yuyue, Jiangsu, China) was used by assistant researchers to measure the BP of the participants. Bedbound participants underwent BP measurements in a recumbent position. The mean DBP and SBP values were calculated using measurements taken twice. Simultaneously, the participants were asked the following question: “Are you suffering from hypertension and have been diagnosed by a hospital?”. According to the guidelines for geriatric hypertension in China, hypertension was defined as an SBP of ≥140 mm Hg, DBP of ≥90 mm Hg, or a standard BP value but self-reported physician diagnosis. The evaluation criterion for hypertension in our study was confirmed in previous study that used the CLHLS data [23].

### Altitude assessment

The key independent variable in our study was residential altitude. For the level of each residential unit (county/district), we obtained the mean altitude from the Digital Elevation Model (DEM) of the National Aeronautics and Space Administration (NASA), and it was accurate to within 30 m. The NASA–DEM is a reprocessing of the Shuttle Radar Topography Mission (SRTM) data with improved accuracy by incorporating auxiliary data from the ASTER GDEM, ICESat GLAS, and PRISM datasets. The altitude value was downloaded from the Google Earth Engine (https://developers.google.cn/earth-engine), and the mean altitude was extracted using ArcGIS 10.6 (ESRI, Redlands, CA, USA). We used the mean residential altitude with a 90-m resolution extracted from the DEM based on the NASA–SRTM3 to assign each unit and examined its association with hypertension in the sensitivity analysis.

### Covariates

In our study, we further adjusted for the following known baseline covariates: age (65–89 years, >89 years), ethnicity (Han Chinese, minority), sex (women, men), residence (rural/town, urban), geographical region (eastern China, central/western China), pension (yes, no), educational attainment (0 year, >0 years), marital status (married/living together, widowed, single/divorced/separated), current smoking status (yes, no), current drinking status (yes, no), current exercise habits (yes, no), high salt intake (yes, no), self-reported heart disease (yes, no), self-reported diabetes (yes, no), and body mass index (BMI) (<18.5 kg/m^2^, 18.5–23.9 kg/m^2^, >23.9 kg/m^2^). We calculated the BMI by dividing body weight (kg) by square of the body height (m^2^), cut-off points for BMI according to the Chinese standard [24].

Given the effects of seasonal and meteorological factors on hypertension incidence reported in previous studies, we collected data on baseline average ambient temperatures in January and annual average precipitation in the county (district) units from the community environment questionnaire, and these were controlled for in the analysis [19]. In addition, we further adjusted for PM_2.5_ concentrations. The data on PM_2.5_ concentrations were satellite-retrieved ground-level concentrations and were obtained from a database developed by the Atmospheric Composition Analysis Group [25]. The database estimated total and compositional ground-level PM_2.5_ mass concentrations worldwide by combining aerosol optical depth retrievals from the NASA MODIS, MISR, and SeaWiFS instruments with the GEOS-Chem chemical transport model, which were subsequently calibrated to regional ground-based observations of both total and compositional masses using geographically weighted regression, with a spatial resolution of 0.01° × 0.01°. This database has a long time series on ground-level PM_2.5_ concentrations in China and has been widely used in previous studies. According to previous research finding [26], the historical one-year average PM_2.5_ concentration in the local units (counties/districts) where the participants resided the year prior to the incident event or censoring was used as an exposure assessment. According to Chinese national ambient air quality standards, PM_2.5_ concentrations were categorized into two categories: <35 µg/m^3^ and ≥35 µg/m^3^.

### Statistical analysis

First, we conducted descriptive statistical analyses to show the distribution of the variables. Categorical and continuous variables were presented as frequencies (n), percentages (%), and means ± standard deviations (SDs), respectively. Continuous variables that were not normally distributed were presented as medians (P_50_) and interquartile ranges (IQRs).

**Fig. 2:**
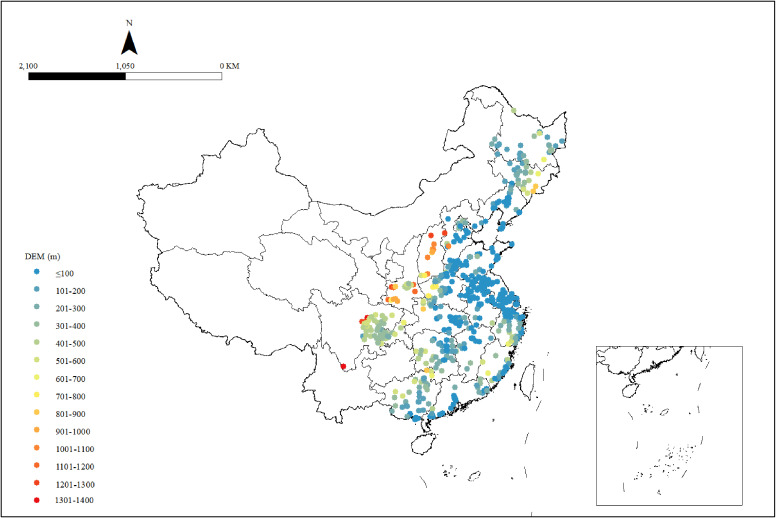
Distribution of altitudes of the residential units assessed in the study. A map was used to visualize the spatial residential altitude distribution of the 613 residential units (county or district) of the 6,548 participants in the study.

We used a map to visualize the spatial residential altitude distribution of the 613 residential units (county or district), as shown in Fig. 2. We also visualized the altitude distributions across China (Fig. S1).

**Fig. 3:**
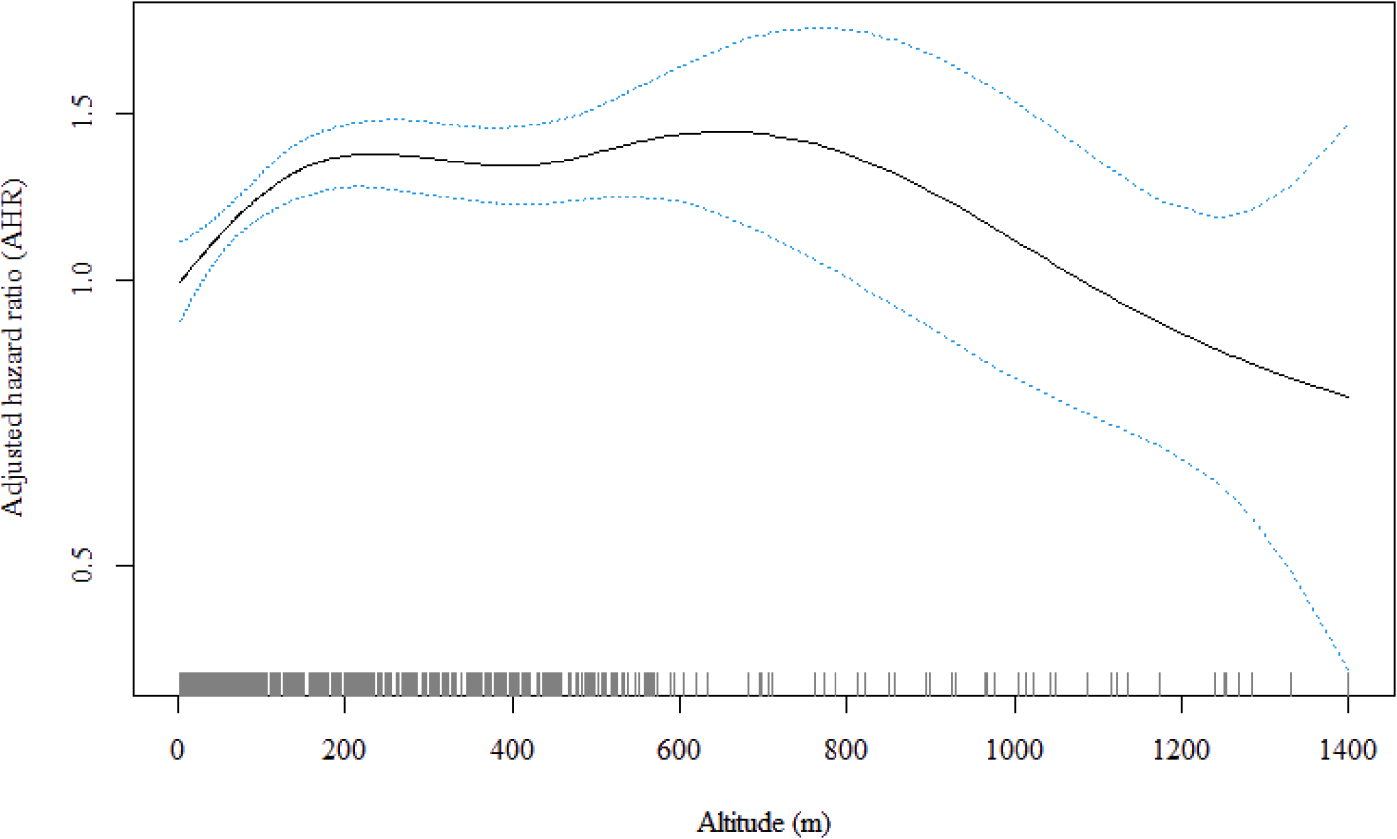
The visible nonlinear association between residential altitude and hypertension risk (df = 4; reference = lowest altitude of 2.8 m). The analysis was adjusted for age, ethnicity, PM_2.5_ concentration, residence, geographical region, sex, current smoking status, current drinking status, current exercise habits, pension, marital status, educational attainment, self-reported diabetes, self-reported heart disease, average ambient temperature in January, average annual precipitation, high salt intake, and body mass index. Abbreviations: df, degree of freedom.

We explored the exposure–response relationship (linear or nonlinear) between altitude and hypertension risk. The Cox proportional hazard models with the penalized spline of different degrees of freedom (dfs) were applied to identify the dose–response association between altitude and hypertension risk after adjusting for age, ethnicity, sex, geographical regions, residence, pension, educational attainment, marital status, current smoking status, current drinking status, current exercise habits, self-reported heart disease, self-reported diabetes, high salt intake, average ambient temperature in January, PM_2.5_ concentration, BMI, and the annual average precipitation. A suitable df value for the analysis depended on the smallest Akaike information criterion value. We also plotted the association between hypertension risk and altitude (Fig. 3) and reported the hypertension risk matched to each altitude (Table S3). According to the shape of the dose–response curve, a statistically significant nonlinear association was observed between altitude and hypertension risk. We then explored the linear association between altitude and hypertension risk. Given the clustering of residential units (county or district), we used the Cox proportional hazard model with a random-effects term for analysis. This analysis was performed after stratifying the data according to the altitude change points that critically determined the shape of the exposure–response curve. In the subgroup analysis, we conducted analyses by age group and sex to determine which group was more vulnerable to altitude exposure. The cross-product term was first used (e.g., altitude * age) to examine the interaction effects of age, sex, and altitude in each stratum determined by the change points from the dose–response curve [21].

In the sensitivity analyses, we used Cox proportional hazard models with a penalized spline of different df values to evaluate the exposure–response association between altitude extracted from NASA–DEM within a 90-m resolution ratio and hypertension incidence in the same way (Fig. S2). We also plotted the exposure–response curve after excluding participants who had changed their addresses (Fig. S3).

Data are presented as hazard ratios (HRs) with 95% confidence intervals (CIs). All the analyses were performed using R v4.0.5 (R Foundation for Statistical Computing, Vienna, Austria), and a two-sided p-value of <0.05 was considered statistically significant.

## Results

### Demographic characteristics of the participants and prevalence of hypertension

Of the 6,548 participants enrolled in the study, the total person-years were 21,255.0, and the mean age was 87.7 years (SD = 11.4). During the whole follow-up period, 1,824 participants were identified as incident hypertension, with an incidence of 8.6 per 100-person years. The detailed demographic characteristics and relevant variables are shown in Table 1. More women (56.0%) were enrolled in the cohort. Table 2 shows the distribution of the participants’ residential altitude variable. The median residential altitude was 130.0 m (IQR = 315.5 m; range = 2.8–1399.7 m) in our study.

**Table 1:** Distribution of the baseline categorical variables of the participants (N = 6548)

**Variables**	**Category**	**N (%)**
Age (years)		
	65–89	3302 (50.4)
	>89	3246 (49.6)
Ethnicity		
	Minority	504 (7.7)
	Han Chinese	6044 (92.3)
Sex		
	Women	3670 (56.0)
	Men	2878 (44.0)
Education attainment (years)		
	0	4070 (62.2)
	>0	2478 (37.8)
Pension		
	No	5373 (82.1)
	Yes	1175 (17.9)
Residence		
	Rural/town	5184 (79.2)
	Urban	1364 (20.8)
Geographical region		
	Central/western	4024 (61.5)
	Eastern	2524 (38.5)
Marital status		
	Single/divorced/separated	82 (1.2)
	Widowed	4432 (67.7)
	Married/living together	2034 (31.1)
Current smoking status		
	No	5422 (82.8)
	Yes	1126 (17.2)
Current drinking status		
	No	5350 (81.7)
	Yes	1198 (18.3)
Current exercise habits		
	No	4714 (72.0)
	Yes	1834 (28.0)
Self-reported diabetes		
	No	6431 (98.2)
	Yes	117 (1.8)
Self-reported heart disease		
	No	6152 (94.0)
	Yes	396 (6.0)
High salt intake		
	No	5497 (83.9)
	Yes	1051 (16.1)
Body mass index (kg/m^2^)		
	>23.9	690 (10.6)
	18.5–23.9	3499 (53.4)
	<18.5	2359 (36.0)
Average temperature in January (°C)		
	≤−9	551 (8.4)
	>−9	5997 (91.6)
Average precipitation (mm)		
	≤800	1849 (28.2)
	>800	4699 (71.8)
PM_2.5_ (µg/m^3^)	<35	1020 (15.6)
	≥35	5528 (84.4)

**Table 2:** Distribution of the altitude of the participants (N = 6548)

	**Mean**	**SD**	**Max.**	**Min.**	**Median**	**IQR**
Residential altitude (m)	223.0	271.0	1399.7	2.8	130.0	315.5

Abbreviations: SD, standard deviation; max., maximum; min., minimum; IQR, interquartile range

### Spatial distribution of the residential units’ (county/district) altitudes

The average residential unit (county/district) altitudes based on the geographical locations of the 613 units are illustrated in Fig. 2. Overall, there is a terrain gradient distribution in China. The altitudes in the western and central regions were relatively higher than those in the east.

### Exposure–response relationships for altitude and hypertension risk

Figure 3 shows the exposure–response relationship for altitude and hypertension risk after adjusting for covariates. The curve was plotted with four dfs, and the p-value for nonlinearity was <0.001. As per the exposure–response curves, the change points of altitude for hypertension risk were 247.1 m, 411.8 m, and 633.9 m (Fig. 3 and Table S3). According to the shape of the curve, there was a monotonic increasing trend in the effects of residential altitude below 247.1 m on hypertension incidence risk. In contrast, the risk of hypertension decreased when participants were exposed to altitudes ranging from 247.2 m to 411.8 m. The risk of hypertension increased over an altitude of 411.9 m, but then plateaued until 633.9 m. Finally, the hypertension risk declined over an altitude of 634.0 m, and the monotone decreased till the end. In the sensitivity analysis, we used an altitude accurate to 90 m to explore the association between altitude and hypertension incidence; in the fully adjusted model, we also detected a nonlinear relationship (Fig. S2). When we excluded participants who changed their addresses, the nonlinear effect of altitude on hypertension incidence remained robust after adjusting for covariates (Fig. S3).

### Linear effects of altitude on hypertension incidence

We further explored the liner association between altitude and hypertension incidence according to the change points (Table 3). Altitudes were divided into four categories: ≤247.1 m, 247.2–411.8 m, 411.9–633.9 m, and ≥634.0 m. In the fully adjusted model, when altitude was ≤247.1 m, higher altitude was significantly associated with an increased risk of hypertension (HR = 1.003; 95% CI = 1.002–1.005; p < 0.05). However, no significant relationship between altitude and risk of hypertension was observed in the other groups. The results did not change after excluding participants who changed their addresses during the investigation (Table 3). Given the availability of the data, we only examined the modification effect of age and sex, and we did not find any significant modification effect in the subgroup analysis (Table S1–S2).

**Table 3:** Association between residential altitude and hypertension incidence among older adults included in this study

	**Change point**			

	**Point 1** **(≤247.1 m)** **n = 4363**	**Point 2** **(247.2–411.8 m)** **n = 962**	**Point 3** **(411.9–633.9 m)** **n = 955**	**Point 4** **(≥634.0 m)** **n = 268**
All participants				
Model 1	**1.002** **(1.001–1.003)**	1.000 (0.997–1.004)	1.001 (0.997–1.005)	0.999 (0.998–1.000)
Model 2	**1.002** **(1.001–1.003)**	1.000 (0.997–1.004)	1.001 (0.997–1.005)	0.999 (0.998–1.000)
Model 3	**1.003** **(1.002–1.005)**	1.002 (0.997–1.007)	1.001 (0.996–1.007)	0.999 (0.969–1.000)
Excluding participants who changed their addresses				
Model 1	**1.002** **(1.001–1.003)**	1.000 (0.997–1.004)	1.001 (0.997–1.005)	0.999 (0.998–1.000)
Model 2	**1.002** **(1.001–1.003)**	1.000 (0.997–1.004)	1.001 (0.997–1.005)	0.999 (0.998–1.000)
Model 3	**1.003** **(1.002–1.005)**	1.002 (0.997–1.007)	1.001 (0.996–1.007)	0.999 (0.997–1.000)

Values indicated in bold were statistically significant.

Model 1: Crude model only including the altitude.

Model 2: Model 1 + further adjustments for age, ethnicity, and sex.

Model 3: Model 2 + further adjustments for age, ethnicity, PM_2.5_ concentration, residence, geographical region, sex, current smoking status, current drinking status, current exercise habits, pension, marital status, educational attainment, self-reported diabetes, self-reported heart disease, average ambient temperature in January, average annual precipitation, high salt intake, and body mass index.

## Discussion

This prospective, long-term, national-scale cohort study provided real-world evidence of a nonlinear association between residential altitude and the incidence of hypertension in older people living at low altitudes. Our research also demonstrates altitude as a possible influencing factor for hypertension. These findings show that a greater awareness of the effects of altitude exposure on hypertension can help healthcare service providers design and implement better preventive strategies for older individuals.

Recently, the risk of hypertension associated with altitude exposure has received increasing attention. In line with previous studies [16, 18], our study also indicated that each unit increase in altitude was a underlying risk factor for hypertension, though with a small effect estimate. However, few studies can be compared with ours because previous investigations were generally conducted in moderate- and high-altitude locations. In addition, many epidemiological investigations have estimated the effect of altitude on BP or hypertension risk based on the hypothesis that the links between altitude exposure and hypertension risk or BP are linear. For instance, Palmer et al. [27] reported that altitude altered BP course during pregnancy, and mean BP rose linearly in participants living in regions at an altitude of >3,100 m. Aryal et al. found that the mean SBP increased by 15.6 mm Hg for every 1,000-m elevation in altitude [28]. Similarly, Mehata et al. reported a significant relationship between altitude and hypertension risk (2% increase per 100 m) in their study conducted among 10,473 Nepalese [29]. Only one cross-sectional study that used national-scale data from the China Hypertension Survey reported that compared with the first reference group (i.e., altitude of <200 m), every 200-m increase in altitude was linked with increased or decreased hypertension risk [8]. The odds ratios (ORs) ranged from 0.96 to 1.37 [8], which was similar to that in our study. Interestingly, the highest OR value was detected when the altitude was between 800 m and 1,000 m rather than >2,200 m, suggesting that the hypertension risk did not monotonically increase with each interval increase in altitude, while this study reported the whole relationship between altitude and hypertension risk was not statistically significant [8]. A clear mechanism for the link between low altitude and hypertension remains unknown. Previous studies illustrated that the adverse effects of high-altitude exposure on hypertension were due to oxygen deficit, cold weather, sympathetic activity, and aging, even though dietary and physical activity patterns might induce hypertension risk [30]. In addition, the effect of altitude on BP may be partly reduced after a long period of exposure to the same altitude [31–33]. Ageing plays an important role in the adverse effects of altitude on hypertension because heart function fails in vascular sclerosis, and BP increase is more frequent in older adults. In addition, poor health conditions and ageing weaken older adults’ abilities to adapt to environmental risk factors [22]. Thus, this complex link between altitude exposure and hypertension risk needs further exploration in future research.

To the best of our knowledge, our study was the first epidemiological analysis to show a significant nonlinear relationship between low altitude and hypertension risk. Previous studies have suggested a possible nonlinear relationship between altitude and hypertension risk. For instance, a study conducted in three Peruvian communities showed that the age-adjusted prevalence of hypertension was lower in the population residing at an altitude of 4,000 m than those in the two reference groups residing at sea level [34]. In a study by Torlasco et al. [35], the authors compared BP changes at moderate and high altitudes. They found that the volunteers' BP measurements started to increase at moderate altitudes (1,500–2,500 m) and showed a pronounced increase at high altitudes. Two studies from Peru and Nepal found that individuals who resided in high-altitude locations had a lower risk of hypertension [9, 36]. We found that the hypertension risk started increasing at the first change point and then slightly fluctuated until the last change point. The risk slowly declined above the last change point (i.e., 634.0 m). Nevertheless, the curve showed that many parts of the confidence intervals did overlap the null. From a risk perspective, areas at an altitude of <300 m are more likely to have high pressure and air density. The natural environment might be more easily destroyed by frequent human activities, leading to high levels of air and noise pollution and smaller areas of green spaces [37, 38]. In comparison, regions with increasing altitude but at <1,000 m (i.e., low altitude) may be suitable for body functions where moderate climatic conditions (temperature, precipitation, humidity, etc.) are ideal [38]. Meanwhile, these areas are generally located in mountainous areas with more vegetation coverage and are farther away from industrial factories. Generally, these areas have good air quality, less pollution, more fresh air, and high negative ion content. In addition, performing more physical activity in these areas can promote blood circulation, improving cardiovascular function. When the altitude is >1,500 m, especially >2,400 m, the estimated risk of hypertension is high due to reduced atmospheric pressure and oxygen in the air, oxygen deficit, and mountain sickness, and this can be accompanied by breathing difficulties [11].

Our study contributes to the currently limited evidence on the effect of low altitude on hypertension, especially given that low-altitude regions have a higher population density and greater environmental risk factors than high-altitude ones. However, several limitations should be acknowledged. First, previous studies have reported that ethnicity affects the difference in tolerance for residential altitude. However, the database used in this study did not include more participants of different ethnicities. As a previous study indicated that Han Chinese individuals had a higher risk of prehypertension than non-Han individuals [39]. Hence, future studies that recruit participants of different ethnicities are warranted. The limited number of included samples in each subgroup prevented us from exploring the modified effects in other subgroups. Future studies should elucidate the effect modification by regions, health status, and outdoor activity patterns. Individuals’ home addresses were not available in the CLHLS because of privacy protection, which precluded us from performing analysis to investigate the associations at an individual’s level. Fourth, our study only focused on altitudes <1,500 m, and thus, the generalizability of the findings was relatively insufficient. Studies with a more heterogeneous sample may help corroborate our findings. Also, data were not available to accurately assess the date of hypertension incidence and use of antihypertensive drugs. Self-reported hypertension diagnosis may have been subject to recall bias. However, in line with a previous study [40], we considered that physicians would prescribe drugs to a person diagnosed with hypertension. Finally, our study only evaluated the links between long-term altitude exposure and the incidence of hypertension. We did not evaluate the link between acute exposure to altitudes and the incidence of hypertension.

In summary, our study findings warrant further research into the associations between altitude exposure and hypertension risk among older adults. This study emphasizes that altitude is an important factor to consider in preventing and controlling hypertension. Promoting awareness about the association between altitude exposure and hypertension risk and adopting practical preventive approaches, such as regular monitoring, early detection, and timely treatment commencement, would be helpful.

## Conclusion

In conclusion, we identified a nonlinear link between altitude exposure and hypertension in this prospective cohort study, including more than 6,000 Chinese elderly living at low altitudes. Healthcare providers should increase awareness of environmental factors to prevent and control hypertension; however, further studies are needed to confirm the generalizability of our study.

## Abbreviations

CLHLS: Chinese Longitudinal Healthy Longevity Survey
DEM: Digital Elevation Model
NASA: National Aeronautics and Space Administration
SD: standard deviation
HR: hazard ratio
CI: confidence interval
CVD: cardiovascular disease
SBP: systolic blood pressure
DBP: diastolic blood pressure
BP: blood pressure
SRTM: Shuttle Radar Topography Mission
df: degree of freedom
IQR: interquartile range
OR: odds ratio
BMI: body mass index
PM: fine particulate matter

## Supplementary information

**Additional file 1:** **Figure S1** The distribution of altitude within 30 m across China. **Figure S2** Sensitivity analysis for the non-linear association between altitude (i.e. altitude accurate to 90 m) and hypertension incidence. **Figure S3** Sensitivity analysis for the non-linear association between altitude and hypertension incidence after excluded participant changed address. **Table S1** The subgroup analysis for the association between altitude and hypertension. **Table S2** The subgroup analysis for the association between sex and hypertension under the influence of altitude over the last change point. **Table S3** The distribution of HR and 95%CI for each altitude based on exposure-response curve.

## Declarations

### Availability of data and material

The data on CLHLS can be down loaded at website: https://sites.duke.edu/centerforaging/programs/chinese-longitudinal-healthy-longevity-survey-clhls/.
