# Supplementary material for: Residential elevation and its effects on hypertension incidence among older adults living at low altitudes: a prospective cohort study
Source: Environ Health Prev Med. 2022 May 3;27:19. doi: 10.1265/ehpm.22-00001 (PMC9251620; doi:10.1265/ehpm.22-00001)
Supplement: Supplementary file 1 — Additional file 1: Figure S1 The distribution of altitude within 30 m across China. Figure S2 Sensitivity analysis for the non-linear association between altitude (i.e. altitude accurate to 90 m) and hypertension incidence. Figure S3 Sensitivity analysis for the non-linear association between altitude and hypertension incidence after excluded participant changed address. Table S1 The subgroup analysis for the association between altitude and hypertension. Table S2 The subgroup analysis for the association between sex and hypertension under the influence of altitude over the last change point. Table S3 The distribution of HR and 95%CI for each altitude based on exposure-response curve. [file ehpm-27-019-s001.pdf]

Supplement materials

Residential elevation and its effects on hypertension incidence among older adults living at low altitudes: a prospective cohort study

Contents

Figure S1 The distribution of altitude within 30 m across China

Figure S2 Sensitivity analysis for the non-linear association between altitude (i.e. altitude accurate to 90 m) and hypertension incidence

Figure S3 Sensitivity analysis for the non-linear association between altitude and hypertension incidence after excluded participant changed address

Table S1 The subgroup analysis for the association between altitude and hypertension

Table S2 The subgroup analysis for the association between sex and hypertension under the influence of altitude over the last change point

Table S3 The distribution of HR and 95%CI for each altitude based on exposure-response curve

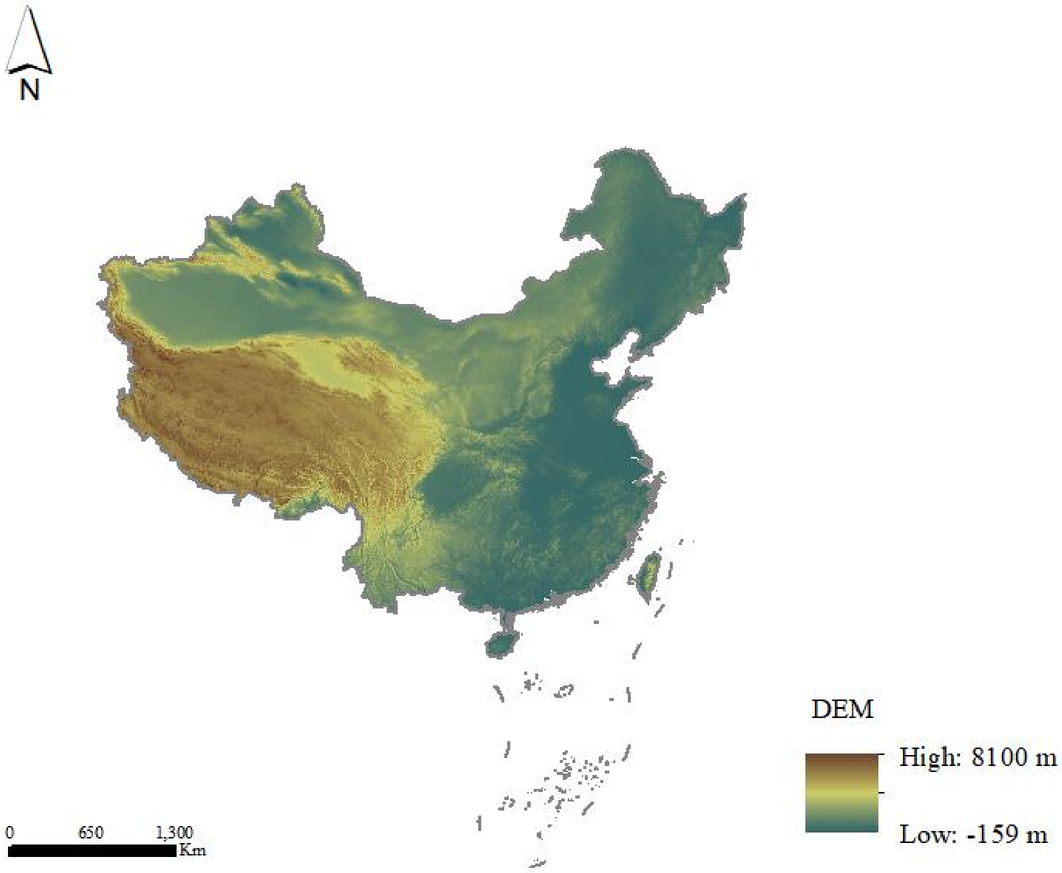

Figure S1 The distribution of altitude within 30m across China

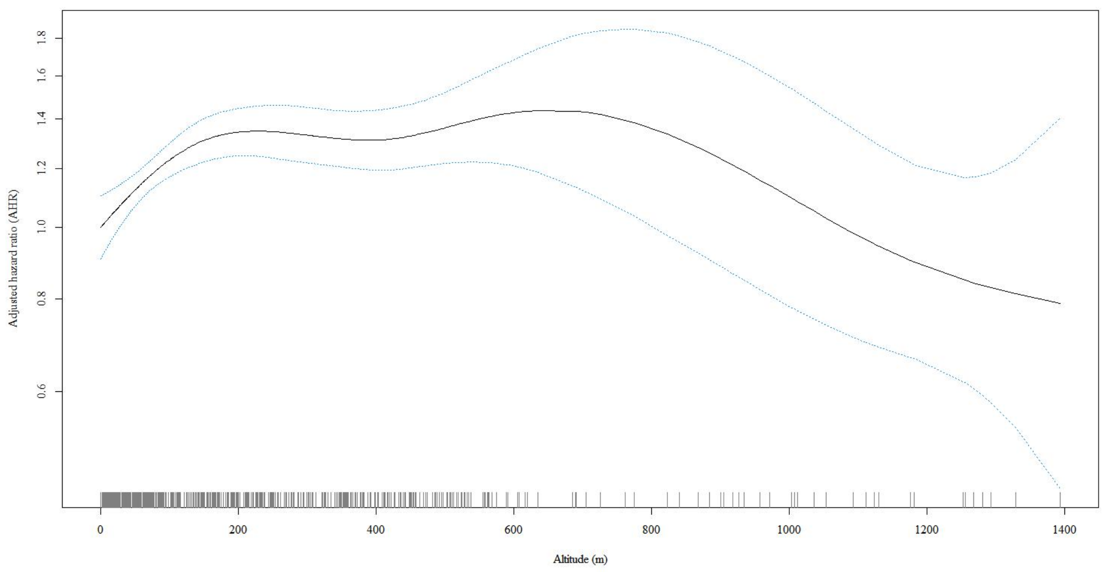

Figure S2 Sensitivity analysis for the non-linear association between altitude (i.e. altitude accurate to 90 m) and hypertension incidence (df=4 and the non-linear P was <0.001 and adjusted for age, ethnicity, $PM_{2.5}$, residence, geographical region, sex, smoking, drinking, exercising, pension, martial status, education attainment, self-reported diabetes, self-reported heart disease, average ambient temperature in the January, annual average of precipitation, high intake of salt and BMI)

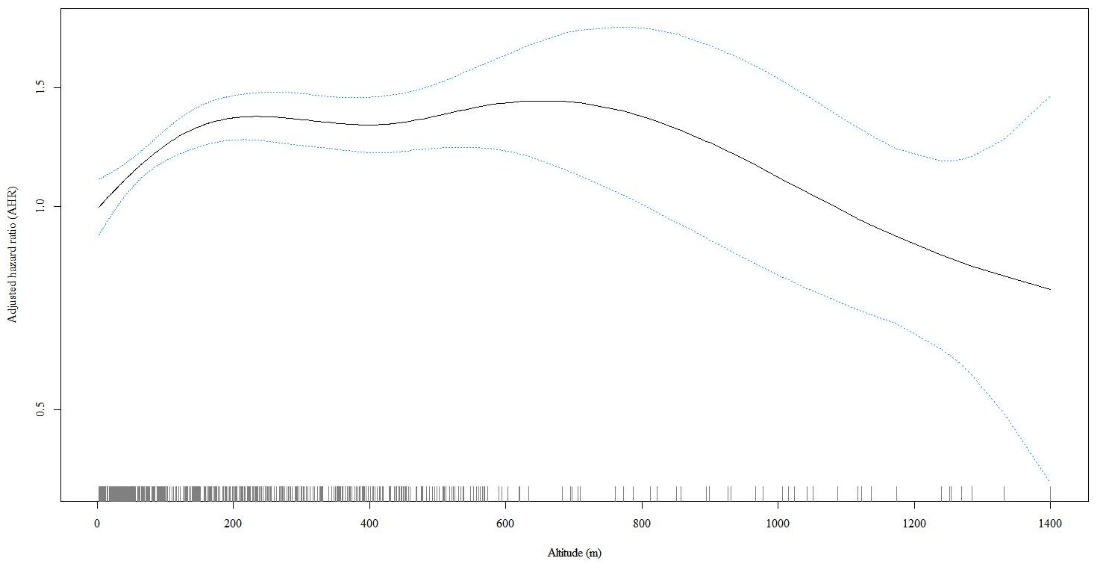

Figure S3 Sensitivity analysis for the non-linear association between altitude and

hypertension incidence after excluded participant changed address (df=4 and the non-linear P was <0.001 and adjusted for age, ethnicity, $PM_{2.5}$, residence, geographical region, sex, smoking, drinking, exercising, pension, martial status, education attainment, self-reported diabetes, self-reported heart disease, average ambient temperature in the January, annual average of precipitation, high intake of salt and BMI)

Table S1 The subgroup analysis for the association between altitude and hypertension

Subgroup	Point 1 (≤247.1 m) n= 4363	Point 2 (247.2-411.8m) n=962	Point 3 (411.9-633.9 m) n=955	Point 4 (≥634.0 m) n=268
	P for interaction effect	P for interaction effect	P for interaction effect	P for interaction effect
Sex				
Women	0.890	0.660	0.560	**0.004**
Men				
Age				
65-89	0.460	0.240	0.270	0.580
>89				

Note:

Values indicated in bold were statistically significant.

The analysis was adjusted for age, ethnicity, $PM_{2.5}$, residence, geographical region, sex, smoking, drinking, exercising, pension, martial status, education attainment, self-reported diabetes, self-reported heart disease, average ambient temperature in the January, annual average of precipitation, high intake of salt and BMI. (unless stratified by the respective factor).

Table S2 The subgroup analysis for the association between sex and hypertension under the influence of altitude over the last change point

Subgroup	Point 4 (≥634.0 m) n=268	P for interaction effect
	Model 1	
Sex		
Women	0.996 (0.990-1.003)	**0.004**
Men	1.001 (0.984-1.004)	

Note:

Values indicated in bold were statistically significant.

The analysis was adjusted for age, ethnicity, $PM_{2.5}$, residence, geographical region, sex, smoking, drinking, exercising, pension, martial status, education attainment, self-reported diabetes, self-reported heart disease, average ambient temperature in the January, annual average of precipitation, high intake of salt and BMI. (unless stratified by the respective factor).

Table S3 The distribution of HR and 95%CI for each altitude based on exposure-response curve

ID	Altitude	HR	Lower limitation for 95%CI	Upper limitation for 95%CI
1	2.845199216	1.000	0.909	1.100
2	3.248210024	1.001	0.911	1.100

3	3.487553648	1.001	0.911	1.100
4	3.494259472	1.001	0.911	1.100
5	3.531293463	1.001	0.911	1.100
6	3.71810089	1.001	0.911	1.100
7	3.735018239	1.001	0.911	1.100
8	3.801242236	1.001	0.912	1.100
9	4.406756757	1.003	0.914	1.101
10	4.802547771	1.004	0.915	1.101
11	4.838137472	1.004	0.915	1.101
12	4.844689379	1.004	0.915	1.101
13	4.868686869	1.004	0.916	1.101
14	4.942273535	1.004	0.916	1.101
15	4.993710692	1.005	0.916	1.102
16	5.045592705	1.005	0.916	1.102
17	5.176912568	1.005	0.917	1.102
18	5.255345144	1.005	0.917	1.102
19	5.261603376	1.005	0.917	1.102
20	5.453493356	1.006	0.918	1.102
21	5.462420382	1.006	0.918	1.102
22	5.58056266	1.006	0.918	1.102
23	5.686940966	1.006	0.919	1.102

24	5.819672131	1.007	0.919	1.103
25	5.852459016	1.007	0.919	1.103
26	5.913793103	1.007	0.919	1.103
27	5.995784148	1.007	0.920	1.103
28	6.081081081	1.007	0.920	1.103
29	6.377919321	1.008	0.921	1.103
30	6.381535039	1.008	0.921	1.103
31	6.527272727	1.008	0.922	1.104
32	6.534351145	1.008	0.922	1.104
33	6.544354839	1.009	0.922	1.104
34	6.921052632	1.009	0.923	1.104
35	6.975	1.010	0.923	1.104
36	7.1	1.010	0.924	1.104
37	7.114336493	1.010	0.924	1.104
38	7.157894737	1.010	0.924	1.104
39	7.23566879	1.010	0.924	1.105
40	7.673684211	1.011	0.926	1.105
41	7.739130435	1.012	0.926	1.105
42	7.780487805	1.012	0.926	1.105
43	7.791666667	1.012	0.926	1.105
44	7.794642857	1.012	0.926	1.105

45	7.90942928	1.012	0.926	1.105
46	7.967054264	1.012	0.927	1.106
47	8.092783505	1.013	0.927	1.106
48	8.563647878	1.014	0.929	1.106
49	8.588235294	1.014	0.929	1.106
50	8.670824053	1.014	0.929	1.107
51	9.459459459	1.016	0.932	1.108
52	9.7	1.017	0.933	1.108
53	9.727272727	1.017	0.933	1.108
54	10.08014911	1.018	0.934	1.109
55	10.23809524	1.018	0.935	1.109
56	10.34482759	1.018	0.935	1.109
57	10.75829384	1.019	0.937	1.109
58	11.33783784	1.021	0.939	1.110
59	11.3627451	1.021	0.939	1.110
60	11.4795082	1.021	0.939	1.110
61	11.5086478	1.021	0.939	1.111
62	11.71581451	1.022	0.940	1.111
63	12.29539295	1.023	0.942	1.112
64	12.3	1.023	0.942	1.112
65	13.13398204	1.026	0.945	1.113

66	14.97375328	1.030	0.951	1.116
67	15.08	1.031	0.952	1.116
68	15.41583055	1.031	0.953	1.116
69	15.88520408	1.033	0.955	1.117
70	16.20643432	1.033	0.956	1.117
71	17.27650728	1.036	0.960	1.119
72	17.62318841	1.037	0.961	1.119
73	17.89473684	1.038	0.962	1.120
74	17.95834564	1.038	0.962	1.120
75	19.16492829	1.041	0.966	1.122
76	19.94074074	1.043	0.969	1.123
77	20.05882353	1.043	0.969	1.123
78	20.17586207	1.044	0.970	1.123
79	20.22996516	1.044	0.970	1.123
80	20.77142857	1.045	0.972	1.124
81	20.93368107	1.046	0.972	1.125
82	20.94357977	1.046	0.972	1.125
83	20.98165138	1.046	0.972	1.125
84	21.81706009	1.048	0.975	1.126
85	21.81845238	1.048	0.975	1.126
86	21.81853282	1.048	0.975	1.126

87	21.94285714	1.048	0.976	1.126
88	22.55279503	1.050	0.978	1.127
89	23.21281646	1.051	0.980	1.128
90	23.29113924	1.052	0.980	1.128
91	23.8317757	1.053	0.982	1.129
92	24.26956522	1.054	0.984	1.130
93	24.28753351	1.054	0.984	1.130
94	24.3994012	1.054	0.984	1.130
95	24.43296089	1.055	0.984	1.130
96	25.38152985	1.057	0.987	1.132
97	25.47451344	1.057	0.988	1.132
98	25.55681818	1.057	0.988	1.132
99	25.82228916	1.058	0.989	1.132
100	26.35294118	1.059	0.991	1.133
101	26.46203474	1.060	0.991	1.133
102	26.55555556	1.060	0.991	1.134
103	26.85714286	1.061	0.992	1.134
104	26.90363036	1.061	0.992	1.134
105	26.93192272	1.061	0.992	1.134
106	27.16883822	1.062	0.993	1.135
107	27.41935484	1.062	0.994	1.135

108	27.68650422	1.063	0.995	1.135
109	27.72795851	1.063	0.995	1.135
110	27.82083333	1.063	0.995	1.136
111	27.850046	1.063	0.996	1.136
112	27.96174445	1.064	0.996	1.136
113	28.23645634	1.064	0.997	1.136
114	28.35826524	1.065	0.997	1.136
115	28.47297297	1.065	0.998	1.137
116	28.49682593	1.065	0.998	1.137
117	28.95901235	1.066	0.999	1.137
118	29.08433735	1.066	1.000	1.138
119	29.14942529	1.067	1.000	1.138
120	29.65549348	1.068	1.001	1.139
121	30.26220302	1.069	1.003	1.140
122	30.55512322	1.070	1.004	1.140
123	30.67881549	1.070	1.005	1.140
124	30.77725118	1.071	1.005	1.141
125	30.77986799	1.071	1.005	1.141
126	30.91139241	1.071	1.006	1.141
127	31.61419753	1.073	1.008	1.142
128	31.63450392	1.073	1.008	1.142

129	31.84090909	1.073	1.009	1.142
130	32.05128205	1.074	1.009	1.143
131	32.07822086	1.074	1.009	1.143
132	32.07962963	1.074	1.009	1.143
133	32.38615752	1.075	1.010	1.143
134	32.54135338	1.075	1.011	1.144
135	33.14912281	1.077	1.013	1.145
136	33.35943775	1.077	1.014	1.145
137	33.69394435	1.078	1.015	1.146
138	34.14778068	1.079	1.016	1.146
139	34.3866588	1.080	1.017	1.147
140	34.41073826	1.080	1.017	1.147
141	34.5326087	1.080	1.017	1.147
142	34.63385949	1.081	1.018	1.147
143	34.64	1.081	1.018	1.147
144	34.76079734	1.081	1.018	1.147
145	34.97727273	1.081	1.019	1.148
146	35.59914712	1.083	1.021	1.149
147	35.61962834	1.083	1.021	1.149
148	36.825	1.086	1.025	1.151
149	37.0873515	1.087	1.025	1.152

150	37.15449202	1.087	1.026	1.152
151	37.26446281	1.087	1.026	1.152
152	37.80453109	1.089	1.028	1.153
153	38.30947776	1.090	1.029	1.154
154	38.30990864	1.090	1.029	1.154
155	38.42666667	1.090	1.030	1.154
156	39.08680556	1.092	1.032	1.155
157	39.55535055	1.093	1.033	1.156
158	39.67857143	1.093	1.034	1.156
159	39.91803279	1.094	1.034	1.157
160	40.58974359	1.096	1.036	1.158
161	40.61184211	1.096	1.036	1.158
162	41.01919643	1.097	1.038	1.159
163	41.24137931	1.097	1.038	1.159
164	41.39019408	1.098	1.039	1.160
165	42.49613402	1.100	1.042	1.162
166	43.1375	1.102	1.044	1.163
167	43.25411882	1.102	1.044	1.163
168	43.27773852	1.102	1.045	1.163
169	43.66305419	1.103	1.046	1.164
170	43.66666667	1.103	1.046	1.164

171	44.05343511	1.104	1.047	1.165
172	44.14908257	1.104	1.047	1.165
173	44.18168168	1.105	1.047	1.165
174	44.19354839	1.105	1.047	1.165
175	44.46693973	1.105	1.048	1.165
176	45.44492441	1.108	1.051	1.167
177	45.54719764	1.108	1.051	1.168
178	45.8907563	1.109	1.052	1.168
179	45.9946714	1.109	1.053	1.168
180	46.51647893	1.110	1.054	1.169
181	46.61491813	1.111	1.054	1.170
182	46.63333333	1.111	1.055	1.170
183	46.79156328	1.111	1.055	1.170
184	47.36660777	1.112	1.057	1.171
185	47.75478348	1.113	1.058	1.172
186	47.76153846	1.113	1.058	1.172
187	47.88235294	1.114	1.058	1.172
188	48.37852761	1.115	1.060	1.173
189	48.54810049	1.115	1.060	1.174
190	49.02564103	1.117	1.061	1.175
191	49.07070707	1.117	1.062	1.175

192	49.33082077	1.117	1.062	1.175
193	50.00501253	1.119	1.064	1.177
194	50.01282051	1.119	1.064	1.177
195	50.32105263	1.120	1.065	1.177
196	52.53726708	1.125	1.071	1.182
197	52.61538462	1.125	1.071	1.182
198	52.78431373	1.126	1.072	1.182
199	53.37313433	1.127	1.074	1.184
200	54.44554455	1.130	1.076	1.186
201	54.50545455	1.130	1.077	1.186
202	55.48595041	1.132	1.079	1.188
203	56.15909091	1.134	1.081	1.190
204	56.23108856	1.134	1.081	1.190
205	56.66496815	1.135	1.082	1.191
206	56.68213457	1.135	1.082	1.191
207	56.82172702	1.136	1.083	1.191
208	59.53781513	1.142	1.090	1.197
209	60.77515192	1.145	1.093	1.200
210	60.95826283	1.146	1.093	1.200
211	61.08695652	1.146	1.094	1.201
212	61.22222222	1.146	1.094	1.201

213	61.4856787	1.147	1.095	1.202
214	62.07167764	1.148	1.096	1.203
215	62.84460695	1.150	1.098	1.205
216	63.17733473	1.151	1.099	1.205
217	63.22568093	1.151	1.099	1.206
218	64.66603053	1.154	1.102	1.209
219	64.80487805	1.155	1.103	1.209
220	64.82793522	1.155	1.103	1.209
221	65.51340996	1.156	1.104	1.211
222	65.71921182	1.157	1.105	1.211
223	66.3783904	1.158	1.106	1.213
224	67.24675325	1.161	1.108	1.215
225	67.2866293	1.161	1.109	1.215
226	68.02173913	1.162	1.110	1.217
227	68.5473301	1.164	1.111	1.218
228	68.77731959	1.164	1.112	1.219
229	68.85585586	1.164	1.112	1.219
230	69.03960396	1.165	1.113	1.219
231	69.18820862	1.165	1.113	1.220
232	69.35347044	1.165	1.113	1.220
233	69.4122738	1.166	1.113	1.220

234	71.04373423	1.169	1.117	1.224
235	71.5295858	1.171	1.118	1.226
236	71.78378378	1.171	1.119	1.226
237	71.94736842	1.172	1.119	1.227
238	72.74333333	1.173	1.121	1.229
239	73.71227621	1.176	1.123	1.231
240	74.87372014	1.178	1.125	1.234
241	74.93972179	1.178	1.125	1.234
242	75	1.179	1.125	1.234
243	75.51028807	1.180	1.126	1.236
244	75.58995327	1.180	1.127	1.236
245	76.07554517	1.181	1.128	1.237
246	76.37168142	1.182	1.128	1.238
247	76.92263056	1.183	1.129	1.239
248	77.58206687	1.184	1.131	1.241
249	80	1.190	1.135	1.247
250	81	1.192	1.137	1.250
251	81.46639511	1.193	1.138	1.251
252	82	1.194	1.139	1.252
253	82.4045977	1.195	1.140	1.253
254	82.47697974	1.195	1.140	1.254

255	83.57446809	1.198	1.142	1.256
256	85.12356575	1.201	1.145	1.260
257	87.64006696	1.207	1.149	1.267
258	88.07	1.208	1.150	1.268
259	88.34782609	1.208	1.150	1.269
260	89.11244019	1.210	1.152	1.271
261	90.44777475	1.213	1.154	1.274
262	90.88103757	1.214	1.155	1.276
263	91.55621302	1.215	1.156	1.277
264	91.68674699	1.215	1.156	1.278
265	92.62273277	1.217	1.158	1.280
266	92.62686567	1.217	1.158	1.280
267	92.72345133	1.218	1.158	1.280
268	93.04047218	1.218	1.158	1.281
269	94.12068966	1.221	1.160	1.284
270	94.27927928	1.221	1.160	1.285
271	96.14446108	1.225	1.163	1.289
272	96.90909091	1.226	1.164	1.291
273	97.5311284	1.228	1.165	1.293
274	98.89555822	1.230	1.167	1.297
275	99.23412204	1.231	1.168	1.298

276	100.1469598	1.233	1.169	1.300
277	100.6465116	1.234	1.170	1.301
278	101.6285714	1.236	1.172	1.304
279	101.7981073	1.236	1.172	1.304
280	103.2797336	1.239	1.174	1.308
281	103.3126551	1.239	1.174	1.308
282	103.7386667	1.240	1.175	1.309
283	106.3028571	1.245	1.178	1.316
284	106.4077381	1.245	1.178	1.316
285	109.4169279	1.251	1.182	1.324
286	110.2728682	1.253	1.184	1.326
287	111.6833058	1.255	1.186	1.329
288	112.139689	1.256	1.186	1.330
289	112.5315985	1.257	1.187	1.331
290	115.1091445	1.262	1.190	1.338
291	116.4085308	1.264	1.192	1.341
292	120.2105751	1.271	1.196	1.350
293	121.9427481	1.274	1.198	1.354
294	126.161408	1.281	1.203	1.363
295	126.2428843	1.281	1.203	1.364
296	128.1057074	1.284	1.205	1.368

297	129.9440129	1.287	1.207	1.372
298	130	1.287	1.208	1.372
299	130.3073868	1.287	1.208	1.372
300	131.2199198	1.289	1.209	1.374
301	132.1823056	1.290	1.210	1.376
302	132.9800664	1.292	1.211	1.378
303	135.7261411	1.296	1.214	1.383
304	136.8295455	1.297	1.215	1.386
305	138.0392157	1.299	1.216	1.388
306	139.5318302	1.301	1.217	1.391
307	140.507716	1.303	1.218	1.392
308	141.4401592	1.304	1.219	1.394
309	142.3175331	1.305	1.220	1.396
310	142.7392694	1.306	1.221	1.397
311	143.0217827	1.306	1.221	1.397
312	143.5429616	1.307	1.221	1.398
313	143.5807494	1.307	1.221	1.398
314	144.0006188	1.307	1.222	1.399
315	144.6719193	1.308	1.222	1.400
316	145.0333333	1.309	1.223	1.401
317	145.4661591	1.309	1.223	1.401

318	146.7623377	1.311	1.224	1.403
319	146.7786885	1.311	1.224	1.403
320	147.98582	1.312	1.226	1.405
321	149.1068702	1.314	1.227	1.407
322	150.8333333	1.316	1.228	1.410
323	150.9583333	1.316	1.228	1.410
324	156.5124782	1.322	1.233	1.418
325	156.5572495	1.322	1.233	1.419
326	156.8739235	1.323	1.233	1.419
327	157.2778001	1.323	1.234	1.420
328	157.3638889	1.323	1.234	1.420
329	157.4763231	1.323	1.234	1.420
330	158.2065262	1.324	1.234	1.421
331	158.8179447	1.325	1.235	1.422
332	160.8628429	1.327	1.236	1.424
333	163.3284314	1.329	1.238	1.428
334	164.2003503	1.330	1.239	1.429
335	165.8053097	1.332	1.240	1.431
336	165.8444613	1.332	1.240	1.431
337	166.1445783	1.332	1.240	1.431
338	166.5224913	1.332	1.240	1.431

339	167.1883959	1.333	1.241	1.432
340	167.9411765	1.334	1.241	1.433
341	170.4608427	1.336	1.243	1.436
342	172.0064935	1.337	1.244	1.438
343	173.258216	1.338	1.245	1.439
344	173.6582556	1.339	1.245	1.439
345	174.2342914	1.339	1.245	1.440
346	174.5027624	1.339	1.245	1.440
347	174.5454545	1.339	1.246	1.440
348	175.5723473	1.340	1.246	1.441
349	177.3679923	1.341	1.247	1.443
350	178.2133252	1.342	1.248	1.444
351	179.5858586	1.343	1.248	1.445
352	179.8137296	1.343	1.248	1.445
353	184.2181231	1.346	1.250	1.449
354	185.4705882	1.347	1.251	1.450
355	188.233731	1.349	1.252	1.452
356	188.31963	1.349	1.252	1.452
357	188.3902538	1.349	1.252	1.452
358	189.528	1.349	1.253	1.453
359	190.3857143	1.350	1.253	1.454

360	191.8085106	1.350	1.253	1.455
361	192.1323529	1.351	1.254	1.455
362	194.3511261	1.352	1.254	1.457
363	195.0406061	1.352	1.254	1.457
364	198.1758578	1.353	1.255	1.459
365	199.0392157	1.354	1.255	1.460
366	199.2324649	1.354	1.256	1.460
367	202.0041237	1.355	1.256	1.462
368	202.2442907	1.355	1.256	1.462
369	202.5207458	1.355	1.256	1.462
370	202.8041401	1.355	1.256	1.462
371	204.783319	1.356	1.257	1.463
372	204.8397891	1.356	1.257	1.463
373	205.2926234	1.356	1.257	1.464
374	208.1600461	1.357	1.257	1.465
375	209.1411765	1.357	1.257	1.466
376	210.4589666	1.358	1.257	1.466
377	211.2533784	1.358	1.257	1.467
378	211.3562329	1.358	1.257	1.467
379	211.3809524	1.358	1.257	1.467
380	213.5842932	1.358	1.257	1.468

381	213.9799235	1.359	1.257	1.468
382	214.0779841	1.359	1.257	1.468
383	214.4262295	1.359	1.257	1.468
384	216.1864734	1.359	1.257	1.469
385	217.8734322	1.359	1.257	1.470
386	220.7078071	1.360	1.257	1.471
387	221.0310049	1.360	1.257	1.471
388	222.0740741	1.360	1.257	1.471
389	222.5994264	1.360	1.257	1.472
390	224.0668919	1.360	1.257	1.472
391	224.9644528	1.360	1.257	1.472
392	225.6391039	1.360	1.257	1.473
393	228.2568389	1.361	1.256	1.474
394	229.7336669	1.361	1.256	1.474
395	230.9860303	1.361	1.256	1.474
396	231.2895894	1.361	1.256	1.474
397	232.2415902	1.361	1.255	1.475
398	233.0223761	1.361	1.255	1.475
399	233.5473684	1.361	1.255	1.475
400	234.7362869	1.361	1.255	1.475
401	235.4400585	1.361	1.255	1.476

402	237.9819597	1.361	1.254	1.476
403	238.0702403	1.361	1.254	1.476
404	240.6135621	1.361	1.253	1.477
405	241.2212389	1.361	1.253	1.477
406	243.1326087	1.361	1.253	1.477
407	247.096745	1.361	1.253	1.477
408	249.5855263	1.360	1.251	1.478
409	249.9152991	1.360	1.251	1.478
410	250.3171753	1.360	1.250	1.478
411	250.6971279	1.360	1.250	1.478
412	251.1842105	1.360	1.250	1.478
413	253.8025794	1.359	1.249	1.479
414	253.9475432	1.359	1.249	1.479
415	254.12	1.359	1.249	1.479
416	254.9749583	1.359	1.249	1.479
417	255.3959075	1.359	1.249	1.479
418	259.9506273	1.358	1.247	1.479
419	263.2084168	1.357	1.246	1.479
420	263.2767892	1.357	1.246	1.479
421	263.5424338	1.357	1.246	1.479
422	267.9002361	1.356	1.244	1.479

423	268.4798913	1.356	1.244	1.479
424	270.7429896	1.356	1.243	1.479
425	271.5177846	1.355	1.243	1.479
426	272.0662139	1.355	1.242	1.478
427	273.7941176	1.355	1.242	1.478
428	275.6680162	1.354	1.241	1.478
429	279.1339869	1.353	1.240	1.477
430	280.7831461	1.353	1.239	1.477
431	282.8496124	1.352	1.238	1.477
432	283.9792899	1.352	1.238	1.476
433	285.6342105	1.351	1.237	1.476
434	291.1369138	1.350	1.235	1.474
435	291.8418079	1.349	1.235	1.474
436	292.0625	1.349	1.235	1.474
437	292.256091	1.349	1.235	1.474
438	292.5551331	1.349	1.235	1.474
439	294.1690196	1.349	1.234	1.474
440	296.0049334	1.348	1.234	1.473
441	299.9289674	1.347	1.232	1.472
442	300.6132979	1.346	1.232	1.471
443	302.6385542	1.346	1.232	1.471

444	303.2638436	1.346	1.231	1.470
445	304.4807122	1.345	1.231	1.470
446	307.2820248	1.344	1.230	1.469
447	307.318038	1.344	1.230	1.469
448	307.5628403	1.344	1.230	1.469
449	309.6695862	1.343	1.229	1.468
450	312.7452229	1.342	1.228	1.467
451	317.2680115	1.341	1.227	1.465
452	319.6876855	1.340	1.226	1.464
453	319.8413223	1.340	1.226	1.464
454	323.806701	1.338	1.225	1.462
455	323.9155251	1.338	1.225	1.462
456	327.0139547	1.337	1.224	1.461
457	327.0351833	1.337	1.224	1.461
458	327.0891365	1.337	1.224	1.461
459	328.1751189	1.337	1.224	1.461
460	329.4569191	1.336	1.223	1.460
461	330.7121771	1.336	1.223	1.460
462	331.3011569	1.336	1.223	1.460
463	339.7014298	1.333	1.220	1.457
464	344.3809524	1.332	1.218	1.455

465	347.909057	1.331	1.217	1.454
466	349.6416443	1.330	1.217	1.454
467	349.929694	1.330	1.217	1.454
468	352.5954198	1.329	1.216	1.453
469	353.4645072	1.329	1.216	1.453
470	354.29555	1.329	1.215	1.453
471	355.0400451	1.329	1.215	1.453
472	355.0656566	1.329	1.215	1.453
473	355.9244151	1.328	1.215	1.453
474	357.0866575	1.328	1.214	1.452
475	358.1309795	1.328	1.214	1.452
476	360.0255213	1.327	1.213	1.452
477	361.031407	1.327	1.213	1.452
478	363.456962	1.327	1.212	1.451
479	367.7341418	1.326	1.211	1.451
480	369.2446461	1.325	1.210	1.451
481	370.7681408	1.325	1.210	1.451
482	371.6290491	1.325	1.210	1.451
483	374.6377462	1.324	1.209	1.451
484	378.6363636	1.324	1.208	1.451
485	380.7945205	1.323	1.207	1.451

486	381.0651526	1.323	1.207	1.451
487	381.2195122	1.323	1.207	1.451
488	382.8501742	1.323	1.207	1.451
489	384.484589	1.323	1.206	1.451
490	384.6299639	1.323	1.206	1.451
491	385.135545	1.323	1.206	1.451
492	385.3634094	1.323	1.206	1.451
493	386.7481633	1.323	1.206	1.451
494	389.09	1.322	1.205	1.451
495	390.8275623	1.322	1.205	1.451
496	391.7764939	1.322	1.204	1.452
497	392.6873823	1.322	1.204	1.452
498	395.373461	1.322	1.204	1.452
499	398.4465541	1.322	1.203	1.453
500	401.4179301	1.322	1.203	1.453
501	404.0222222	1.322	1.202	1.454
502	404.3027454	1.322	1.202	1.454
503	405.3482587	1.322	1.202	1.454
504	407.9126383	1.322	1.202	1.455
505	411.7675676	1.322	1.202	1.455
506	414.2548765	1.323	1.202	1.457

507	414.6619971	1.323	1.202	1.457
508	415.331124	1.323	1.202	1.457
509	419.2857143	1.324	1.202	1.458
510	420.1408935	1.324	1.202	1.459
511	420.1455175	1.324	1.202	1.459
512	420.2739362	1.324	1.202	1.459
513	429.4386694	1.326	1.203	1.462
514	429.8384401	1.326	1.203	1.463
515	430.6499705	1.327	1.203	1.463
516	436.1776316	1.328	1.204	1.466
517	438.0175342	1.329	1.204	1.467
518	438.607717	1.329	1.205	1.467
519	439.9182156	1.330	1.205	1.468
520	442.790454	1.331	1.206	1.469
521	443.7756926	1.331	1.206	1.470
522	446.0257206	1.332	1.207	1.471
523	447.6613027	1.333	1.207	1.472
524	449.5655356	1.334	1.208	1.473
525	451.5609756	1.335	1.208	1.475
526	451.9052347	1.335	1.208	1.475
527	452.5699043	1.335	1.208	1.475

528	454.5085008	1.336	1.209	1.477
529	457.0690953	1.338	1.210	1.479
530	459.1746442	1.339	1.211	1.480
531	467.648855	1.343	1.213	1.487
532	467.7246732	1.343	1.213	1.487
533	469.3947507	1.344	1.214	1.489
534	475.5326004	1.348	1.216	1.495
535	477.6599351	1.349	1.216	1.497
536	477.698324	1.349	1.216	1.497
537	483.305296	1.353	1.218	1.503
538	487.5956284	1.356	1.219	1.508
539	488.2471483	1.356	1.219	1.509
540	492.3417722	1.359	1.220	1.514
541	492.6410256	1.359	1.220	1.514
542	495.7021277	1.361	1.221	1.518
543	498.2577519	1.363	1.222	1.521
544	502.1300813	1.366	1.222	1.526
545	507.3969466	1.370	1.223	1.533
546	508.9861111	1.371	1.224	1.536
547	509.6716418	1.371	1.224	1.537
548	510.0967742	1.372	1.224	1.537

549	510.1649485	1.372	1.224	1.537
550	512.1818182	1.373	1.224	1.540
551	517.0233372	1.377	1.225	1.547
552	520.8981023	1.379	1.225	1.553
553	522.5843072	1.381	1.225	1.556
554	524.7438167	1.382	1.225	1.559
555	530.477431	1.386	1.225	1.568
556	531.1078125	1.387	1.225	1.569
557	533.5170213	1.388	1.225	1.573
558	534.2808456	1.389	1.225	1.574
559	537.3431288	1.391	1.225	1.579
560	538.862963	1.392	1.225	1.581
561	547.9458599	1.398	1.225	1.596
562	552.2576177	1.401	1.224	1.603
563	557.3462695	1.404	1.224	1.611
564	559.8021454	1.406	1.223	1.615
565	561.9634146	1.407	1.223	1.619
566	565.276084	1.409	1.222	1.624
567	566.0334928	1.409	1.222	1.625
568	567.9087098	1.410	1.222	1.628
569	569.0963303	1.411	1.221	1.630

570	574.0740741	1.414	1.220	1.638
571	589.3564174	1.421	1.215	1.662
572	593.7225058	1.423	1.213	1.669
573	603.5868748	1.427	1.208	1.685
574	619.3154472	1.432	1.198	1.710
575	620.3318753	1.432	1.197	1.712
576	633.9206642	1.434	1.187	1.733
577	682.6770833	1.433	1.140	1.803
578	695.1083009	1.430	1.127	1.816
579	696.8550955	1.430	1.125	1.818
580	706.0568116	1.427	1.115	1.826
581	709.6154478	1.425	1.111	1.828
582	760.840367	1.395	1.054	1.847
583	773.1638907	1.386	1.040	1.846
584	787.2851296	1.373	1.023	1.844
585	812.0101652	1.349	0.993	1.834
586	822.2960358	1.339	0.980	1.827
587	850.7449848	1.306	0.947	1.801
588	856.8506289	1.299	0.940	1.794
589	894.7650303	1.250	0.898	1.741
590	899.3159527	1.244	0.893	1.733

591	926.0066288	1.208	0.865	1.689
592	930.4013378	1.202	0.860	1.681
593	966.2413793	1.153	0.823	1.616
594	967.3501715	1.151	0.822	1.614
595	977.5196078	1.137	0.812	1.594
596	1006.219144	1.098	0.786	1.535
597	1014.485915	1.087	0.779	1.517
598	1023.694499	1.075	0.771	1.498
599	1041.966216	1.051	0.757	1.459
600	1050.744069	1.039	0.750	1.440
601	1086.864078	0.994	0.723	1.367
602	1116.203194	0.961	0.704	1.311
603	1122.132404	0.954	0.701	1.300
604	1136.506056	0.939	0.692	1.274
605	1173.680913	0.903	0.670	1.216
606	1173.68879	0.903	0.670	1.216
607	1240.031933	0.847	0.615	1.168
608	1251.50625	0.839	0.602	1.168
609	1254.302954	0.837	0.599	1.169
610	1268.669048	0.827	0.582	1.175
611	1284.481314	0.817	0.562	1.187

612	1331.827862	0.789	0.494	1.259
613	1399.65625	0.754	0.389	1.459
